# Latent class analysis does not support the existence of Rome IV functional bowel disorders as discrete entities

**DOI:** 10.1111/nmo.14391

**Published:** 2022-05-09

**Authors:** Christopher J. Black, Lesley A. Houghton, Alexander C. Ford

**Affiliations:** ^1^ Leeds Institute of Medical Research at St. James’s University of Leeds Leeds UK; ^2^ Leeds Gastroenterology Institute St. James’s University Hospital Leeds UK

**Keywords:** functional bowel disorders, latent class analysis, mood, Rome IV, somatization, subgrouping

## Abstract

**Background:**

Previously, we used latent class analysis (LCA) to identify novel subgroups in people with irritable bowel syndrome (IBS). There are four other functional bowel disorders that, although characterized as discrete disorders, overlap considerably with, and fluctuate to, IBS. These might instead be conceptualized as a milder form of IBS. We explored this hypothesis using LCA in a cohort of people with non‐IBS functional bowel disorders.

**Methods:**

We collected demographic, symptom, and psychological health data from 1375 adults in the community who self‐identified as having IBS and identified individuals meeting Rome IV criteria for any non‐IBS functional bowel disorder. We performed LCA to identify specific subgroups (clusters). We followed participants up at 12 months to reassess gastrointestinal and psychological heath and also gather data about healthcare utilization and impact of symptoms.

**Key results:**

811 people met Rome IV criteria for IBS and 558 Rome IV criteria for another functional bowel disorder (76 (5.5%) functional constipation; 198 (14.5%) functional diarrhea; 129 (9.5%) functional abdominal bloating or distension; and 155 (11.4%) unspecified functional bowel disorder). LCA in these 558 people identified five clusters defined by a combination of gastrointestinal symptoms and the extent of psychological co‐morbidity. However, correlation between these clusters and the Rome IV functional bowel disorder diagnoses was poor and 75% of people were classified as having mild IBS using our previous IBS‐derived model. By 12 months, one‐third of people had fluctuated and met criteria for IBS. Clusters with high psychological burden had a poorer prognosis, with higher rates of medical consultation, medication use, and greater impact of symptoms on daily life.

**Conclusions and inferences:**

The functional bowel disorders may be better characterized as a spectrum of IBS rather than separate disorders. Adopting this pragmatic stance may help to simply diagnosis, treatment, and recruitment of patients to research trials.

## Graphical abstract

Among 558 people with a Rome IV functional bowel disorder, excluding IBS, latent class analysis identified five unique clusters characterized by a profile of gastrointestinal symptoms, extra‐intestinal symptoms, and mood. However, correlation between these clusters and the Rome IV functional bowel disorder diagnoses was poor, and 75% of people were classified as having mild IBS using our previous IBS‐derived model. The functional bowel disorders may be better characterized as a spectrum of IBS rather than separate disorders.

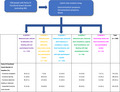

**Abbreviations**
ANOVA: analysis of variance
BIC(LL): Bayesian information criterion of the log‐likelihood
CPSS: Cohen perceived stress scale
HADS: hospital anxiety and depression scale
IBS: irritable bowel syndrome
IBS‐C: IBS with constipation
IBS‐D: IBS with diarrhea
LCA: latent class analysis
PHQ‐12: patient health questionnaire‐12
VSI: visceral sensitivity index

**Key Points**

Our previous work identified novel subgroups among people with irritable bowel syndrome (IBS) using latent class analysis (LCA). There are four other functional bowel disorders, characterized as discrete conditions, but which overlap with, and fluctuate to, IBS.

- This study used LCA in a large cohort of people with non‐IBS functional bowel disorders and identified five unique clusters based on gastrointestinal symptoms, extra‐intestinal symptoms, and mood.
- Correlation between these clusters and the Rome IV functional bowel disorder diagnoses was poor and 75% of people were classified as having mild IBS based on our previous IBS‐derived subgrouping model.
- The functional bowel disorders may be better characterized as a spectrum of IBS rather than separate disorders.

## 1 INTRODUCTION

Functional bowel disorders are a group of conditions that include irritable bowel syndrome (IBS), functional constipation, functional diarrhea, functional abdominal bloating or distension, and unspecified functional bowel disorder.^1^ Functional gastrointestinal disorders such as these have been re‐termed disorders of gut‐brain interaction,^2^ due to the integral role of central nervous system dysregulation of modulation of gut signaling and motor function in symptom generation and persistence. Functional bowel disorders are some of the commonest of these disorders of gut‐brain interaction, with IBS and functional constipation estimated to affect between 5% and 10% of individuals globally.^3^, ^4^ These conditions have a considerable economic impact on societies,^5^, ^6^ and lead to substantial impairments in quality of life and social functioning in patients.^7^

Multinational global surveys conducted using state‐of‐the‐art, standardized, methodology reveal that between 30% and 35% of individuals report symptoms compatible with a functional bowel disorder at any one point in time.^7^, ^8^ However, the criteria used to define these conditions have evolved with successive iterations of the Rome criteria,^9^, ^10^, ^11^ meaning that, although the overall prevalence of functional bowel disorders as a group has remained stable, the relative frequency of each has changed. As the criteria used to define the presence of IBS have become more restrictive, fewer people now meet criteria for this condition. In moving from the Rome III definition of IBS to Rome IV, prevalence in the general population halved from 9% to 4.6%.^7^ At the same time, the prevalence of unspecified functional bowel disorder according to Rome IV criteria in these surveys is now estimated to be between 9% and 11%.^7^, ^8^

There are some limitations of the current classification system for functional bowel disorders. First, these conditions, when defined according to the Rome IV criteria, are not stable. In a longitudinal follow‐up study, almost one‐third of individual with Rome IV IBS fluctuated to another functional bowel disorder at 12 months.^12^ Second, the only licensed treatments available for functional bowel disorders are those for IBS or functional constipation. For patients with any of the other three conditions, there is a lack of clear evidence as to how best to treat them. Finally, unspecified functional bowel disorder may now be the most prevalent of these conditions, but it is uncertain what this encompasses, or whether it even exists, given it consists entirely of people who do not meet criteria for any of the other four functional bowel disorders.

Previously, we have used a statistical technique called latent class analysis (LCA), to subgroup people with Rome IV‐defined IBS. LCA is a method of mathematical modeling which can identify unobserved clusters, or latent classes, within observed multivariate data.^13^ We demonstrated that people with IBS can be divided into seven distinct and reproducible clusters.^14^ These were characterized by a pattern of gastrointestinal symptoms (predominantly diarrhea‐related, predominantly constipation‐related, or mixed symptoms) further differentiated by the presence or absence of abdominal pain not relieved by defecation, and by the presence or absence of high levels of both extra‐intestinal symptom reporting and psychological co‐morbidity.

Our previous study recruited people who self‐identified with IBS,^14^ but we restricted our analysis to those individuals who met Rome IV criteria for IBS. The remaining participants met Rome criteria for a different functional bowel disorder, but were comparable with the IBS cohort in terms of demographics. Given the fluctuation between functional bowel disorders, and the lack of stability over time, we postulated that applying LCA to patients with the remaining four functional bowel disorders would not yield the discrete groups of patients that are currently described by the classification system. Instead, we hypothesized that these four functional bowel disorders perhaps represent the milder end of the IBS spectrum, and that they should be managed accordingly. We therefore applied LCA to a cohort of individuals with lower gastrointestinal symptoms, meeting Rome IV criteria for functional constipation, functional diarrhea, functional abdominal bloating or distension, or unspecified functional bowel disorder to examine the clusters derived and followed these up longitudinally to examine their natural history.

## 2 METHODS

### 2.1 Participants and setting

Participants were individuals who self‐identified as having IBS registered with three organizations in the UK, and who agreed to participate in a previous study published elsewhere.^14^, ^15^ These were the IBS network, the registered charity for people living with the condition, TalkHealth, an online social health community providing information about various medical conditions, and ContactMe‐IBS, a dedicated research register allowing individuals with IBS to participate in research. Those aged ≥18 years were eligible with no exclusions to participation, other than an inability to understand written English. We invited individuals, via email and post, between December 2017 and December 2018, directing them to a study information leaflet. Those interested completed an online questionnaire. We stored all responses securely in an online database. We did not provide any financial incentives to participate. We sent identical follow‐up questionnaires to all participants 12 months later, using the same methods. The University of Leeds research ethics committee approved both baseline and follow‐up studies in November 2017. We have reported data from the individuals who met Rome IV or III criteria for IBS in this cohort previously.^12^, ^16^, ^17^, ^18^, ^19^

### 2.2 Data collection and synthesis

#### 2.2.1 Baseline data collection

We collected baseline demographic data and asked respondents to state whether they had seen a primary care physician or a gastroenterologist about their gastrointestinal symptoms. We captured lower gastrointestinal symptom data at baseline using the Rome IV questionnaire.^20^ Among those individuals who did not meet the Rome IV criteria for IBS at baseline, we used the scoring algorithms proposed for use with the Rome IV questionnaire to assign presence or absence of the four other Rome IV‐defined functional bowel disorders. We measured the impact of gastrointestinal symptoms at baseline in terms of the proportion of time that they limited normal daily activities, according to the Rome IV questionnaire,^20^ and dichotomized this at a threshold of interference with daily activities ≥50% of the time.

We collected anxiety and depression data via the hospital anxiety and depression scale (HADS),^21^ with a total score ranging from a minimum of 0 to a maximum of 21 for either anxiety or depression. A score ≤7 is considered normal, 8–10 borderline abnormal, and ≥11 abnormal. We collected extra‐intestinal symptom data via the patient health questionnaire‐12 (PHQ‐12),^22^ derived from the validated PHQ‐15.^23^ The total PHQ‐12 score ranges from a minimum of 0 to a maximum of 24. We categorized severity into high (total PHQ‐12 ≥13), medium (8–12), low (4–7), or minimal (≤3).

We utilized the 15‐item visceral sensitivity index (VSI),^24^ which measures gastrointestinal symptom‐specific anxiety. Replies to each item are provided on a six‐point scale from “strongly disagree” (scored as 0) to “strongly agree” (scored as 5). There are no validated cut offs to define levels of gastrointestinal symptom‐specific anxiety. We therefore divided these data into equally sized tertiles across the entire cohort of participants. We used the 10‐item version of the Cohen perceived stress scale (CPSS) to assess perceived stress. This is derived from the original 14‐item instrument,^25^ and is psychometrically reliable and comparable with it.^26^ It measures experience of stress in the previous month. Again, with no validated cut offs to define levels of perceived stress, we divided data into tertiles across the entire cohort.

### 2.3 12‐month follow‐up data collection

We asked participants to state whether they had seen a primary care physician or gastroenterologist about their gastrointestinal symptoms in the 12 months since study entry, and whether they had commenced any new treatments (dietary, drugs, and/or psychological) for their symptoms since study entry. We measured impact of gastrointestinal symptoms, as well as mood and extra‐intestinal symptoms using the same instruments as at baseline. Finally, we assigned presence of IBS at 12 months according to the Rome IV questionnaire.^20^

### 2.4 Statistical analysis

We performed LCA using LatentGOLD (version 5.1 Statistical Innovations, Belmont, MA, USA) only in individuals with functional constipation, functional diarrhea, functional abdominal bloating or distension, or unspecified functional bowel disorder.^27^ This is a method of structural equation modeling to identify unobserved groups, or latent classes, within the observed multivariate data.^13^ It postulates a statistical model for the population from which the data sample is obtained, assuming a mixture of underlying probability distributions generates the data.^28^ This approach is referred to as model‐based clustering. It is flexible, allowing a range of variable types to be incorporated within the same model, and iterative as, for any given number of clusters, multiple solutions are evaluated to determine the best output.^28^ The best fit of the model, and the optimum number of clusters, is determined by robust statistical criteria.^29^ For this purpose, we used the Bayesian information criterion of the log‐likelihood (BIC(LL)), selecting the cluster solution with the lowest BIC(LL) value as the one that best fit the data. Details of the variables used in the model are provided in Supplementary Table 1.

**TABLE 1:** Characteristics of the five clusters observed among 558 individuals with a Rome IV functional bowel disorder

	Cluster 1 Abdominal pain relieved by defecation and bloating with low psychological burden (*n* = 170)	Cluster 2 Abdominal pain relieved by defecation, loose/watery stools, and urgency with low psychological burden (*n* = 139)	Cluster 3 Abdominal pain, abnormal stool frequency or consistency, urgency, fecal incontinence, and bloating with high psychological burden (*n* = 118)	Cluster 4 Abdominal pain, abnormal stool consistency, urgency, fecal incontinence, and bloating with high psychological burden (*n* = 90)	Cluster 5 Hard/lumpy stools and fecal incontinence with low psychological burden (*n* = 41)	Total (*n* = 558)	*P* value
Mean age (SD)	50.6 (15.5)	54.2 (14.4)	48.5 (15.6)	48.3 (13.6)	63.7 (14.5)	51.6 (15.4)	<0.001
Female (%)	130 (76.5)	114 (82.0)	103 (87.3)	79 (87.8)	29 (70.7)	455 (81.5)	0.026
Symptoms limiting activities ≥50% of the time at baseline (%)	49 (28.8)	83 (59.7)	68 (57.6)	51 (56.7)	2 (4.9)	253 (45.3)	<0.001
Seen a primary care physician with symptoms at baseline (%)	153 (90.0)	133 (95.7)	111 (94.1)	85 (94.4)	36 (87.8)	518 (92.8)	0.21
Seen a gastroenterologist with symptoms at baseline (%)	74 (43.5)	74 (53.2)	59 (50.0)	62 (68.9)	23 (56.1)	292 (52.3)	0.003
High VSI scores at baseline (%)	13 (7.6)	27 (19.4)	36 (30.5)	36 (40.0)	1 (2.4)	113 (20.3)	<0.001
High CPSS scores at baseline (%)	10 (5.9)	16 (11.5)	35 (29.7)	22 (24.4)	1 (2.4)	84 (15.1)	<0.001
Rome IV functional bowel disorder at baseline (%)							
Functional constipation	36 (21.2)	7 (5.0)	12 (10.2)	14 (15.6)	7 (17.1)	76 (13.6)	
Functional diarrhea	51 (30.0)	80 (57.6)	39 (33.1)	14 (15.6)	14 (34.1)	198 (35.5)	
Functional abdominal bloating or distension	46 (27.1)	23 (16.5)	31 (26.3)	23 (25.6)	6 (14.6)	129 (23.1)	
Unspecified functional bowel disorder	37 (21.8)	29 (20.9)	36 (30.5)	39 (43.3)	14 (34.1)	155 (27.8)	<0.001
IBS cluster at baseline (%)							
Cluster 1	0 (0)	72 (51.8)	8 (6.8)	0 (0)	0 (0)	80 (14.3)	
Cluster 2	0 (0)	0 (0)	52 (44.1)	71 (78.9)	0 (0)	123 (22.0)	
Cluster 3	162 (95.3)	52 (37.4)	27 (22.9)	17 (18.9)	41 (100)	299 (53.6)	
Cluster 4	0 (0)	5 (3.6)	16 (13.6)	0 (0)	0 (0)	21 (3.8)	
Cluster 5	0 (0)	0 (0)	3 (2.5)	0 (0)	0 (0)	3 (0.5)	
Cluster 6	0 (0)	0 (0)	3 (2.5)	0 (0)	0 (0)	3 (0.5)	
Cluster 7	8 (4.7)	10 (7.2)	9 (7.6)	2 (2.2)	0 (0)	29 (5.2)	<0.001

* *p* value for one‐way ANOVA for continuous data and Pearson *χ* ^2^ for comparison of categorical data.

We drew a radar plot, using *z*‐values for each variable, for each of the clusters. We calculated these by adjusting the cluster mean for each variable to the cohort mean and standard deviation for that variable. We then compared radar plots by visual inspection, describing the characteristics of each cluster.

We compared baseline characteristics of individuals, such as age, sex, impact of symptoms, consultation behavior, and gastrointestinal symptom‐specific anxiety and perceived stress levels in each cluster, as well as applying the original LCA clusters from the participants with Rome IV‐defined IBS (Box 1 and Figure S1),^14^ to examine how these were distributed across the individuals who had one of the four other Rome IV functional bowel disorders according to the new clusters derived in this study. We also compared 12‐month data including impact of symptoms, consultation behavior, commencement of new treatments, mood, extra‐intestinal symptoms, and whether participants met Rome IV criteria for IBS at follow‐up according to cluster. We compared categorical data between clusters using a chi‐squared test and continuous variables using a one‐way analysis of variance (ANOVA) test. Due to multiple comparisons, we considered a 2‐tailed *p* value of <0.01 as statistically significant for these analyses, which we performed using SPSS for Windows (version 24.0 SPSS Inc., Chicago, IL, USA).

BOX 1 **Descriptions of the seven clusters identified at baseline among 811 individuals with Rome IV‐defined IBS.**

**Table:** 

Cluster 1: Diarrhea and urgency with low psychological burden.
Cluster 2: Low overall gastrointestinal symptom severity with high psychological burden.
Cluster 3: Low overall gastrointestinal symptom severity with low psychological burden.
Cluster 4: Diarrhea, abdominal pain, and urgency with high psychological burden.
Cluster 5: Constipation, abdominal pain, and bloating with high psychological burden.
Cluster 6: High overall gastrointestinal symptom severity with high psychological burden.
Cluster 7: Constipation and bloating with low psychological burden.

Note: The terms “high” or “low” are made with reference to the adjustment of variables with respect to the cohort average for each variable using *z*‐scores. More detail is provided in our previous paper, and the radar plots for these clusters are provided in Figure S1.^14^

## 3 RESULTS

We recruited 1375 individuals into the study. The mean age of subjects was 49.2 years (range 18–86 years), 1157 (84.1%) were female, and 1293 (94.0%) were White Caucasian. The 811 (59.0%) participants with Rome IV IBS were excluded from this analysis, and a further six individuals provided incomplete data. Of the remaining 558 subjects, 76 (5.5%) met Rome IV criteria for functional constipation, 198 (14.5%) functional diarrhea, 129 (9.5%) functional abdominal bloating or distension, and 155 (11.4%) unspecified functional bowel disorder. They had a mean age of 51.6 years (range 19–86 years) and 455 (81.5%) were female.

### 3.1 Latent class analysis in the individuals with Rome IV‐defined functional bowel disorders

**FIGURE 1:**
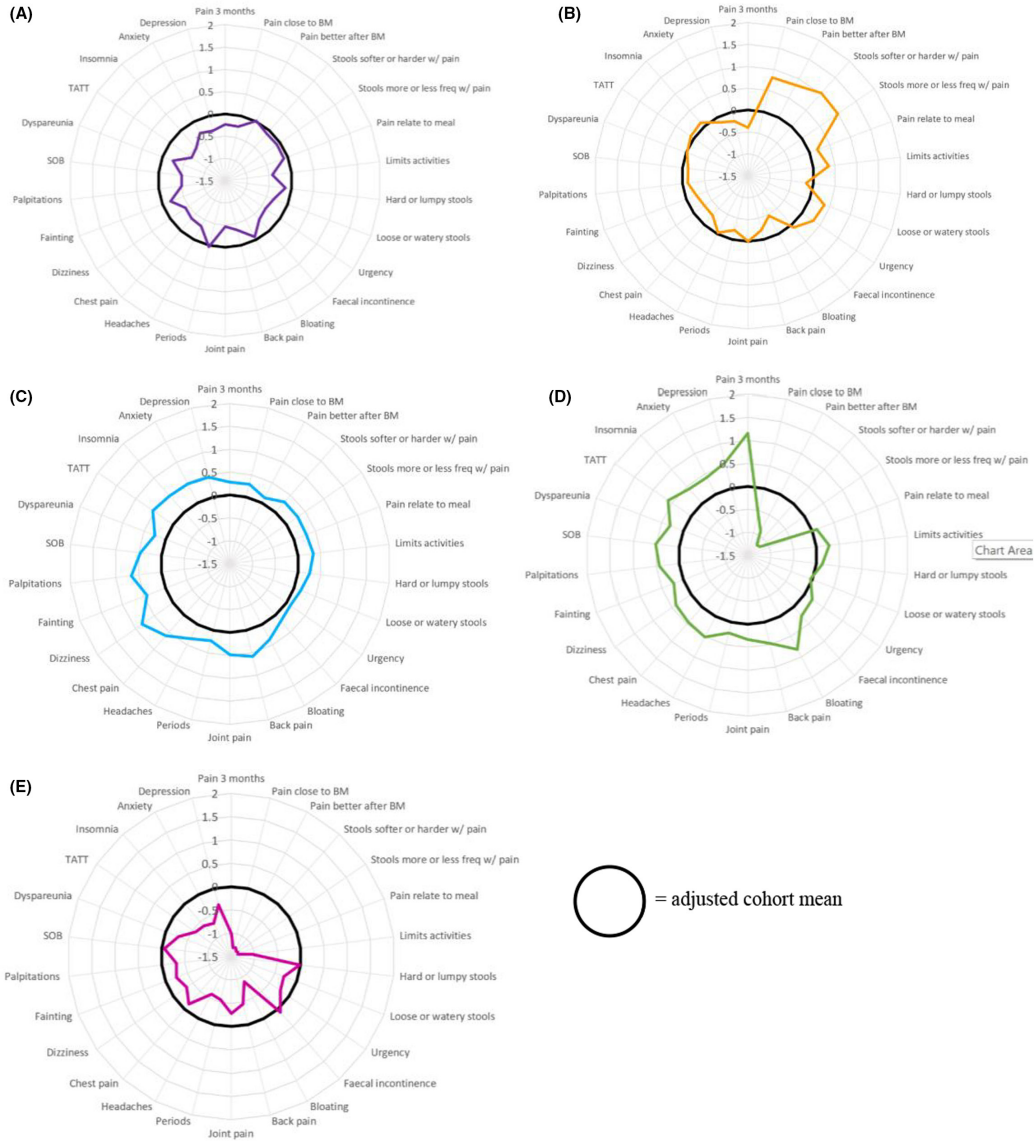
Profiles of the Five Latent Class Clusters Identified in the non‐IBS Rome IV Functional Bowel Disorder Cohort. (A) Cluster 1: Abdominal pain relieved by defecation and bloating with low psychological burden. (B) Cluster 2: Abdominal pain relieved by defecation, loose/watery stools, urgency, and fecal incontinence with low psychological burden. (C) Cluster 3: Abdominal pain, abnormal stool frequency or consistency, urgency, fecal incontinence, and bloating with high psychological burden. (D) Cluster 4: Abdominal pain, abnormal stool consistency, urgency, fecal incontinence, and bloating with high psychological burden. (E) Cluster 5: Hard/lumpy stools and fecal incontinence with low psychological burden. BM: bowel movement; GI: gastrointestinal; SOB: shortness of breath; TATT: tired all the time

The best LCA solution was achieved with five clusters, as indicated by the lowest value of the BIC(LL) (Figure S2). Radar plots for each of these clusters are presented in Figure 1. Two clusters were characterized by below‐average scores for all gastrointestinal symptoms, other than abdominal pain relieved by defecation and bloating (Figure 1A) or hard/lumpy stools and fecal incontinence (Figure 1E), and below‐average scores for extra‐intestinal and mood‐related symptoms. Similarly, another two of the clusters were characterized by well above‐average scores for gastrointestinal symptoms including abdominal pain, abnormal stool frequency or consistency, urgency, fecal incontinence, and bloating (Figure 1C) or abdominal pain, abnormal stool consistency, urgency, fecal incontinence, and bloating (Figure 1D), and well above‐average scores for extra‐intestinal and mood‐related symptoms. The final cluster (Figure 1B) was characterized by above‐average scores for abdominal pain relieved by defecation, loose/watery stools, urgency, and fecal incontinence with below‐average scores for extra‐intestinal and mood‐related symptoms.

### 3.2 Characteristics of the different clusters

The characteristics of the five clusters are shown in Table 1. There was a difference in mean age between clusters, with those in cluster 5 being significantly older (*p *< 0.001). Proportions of people reporting impact of symptoms on activities of daily living ≥50% of the time was significantly greater in clusters 2, 3, and 4 (*p *< 0.001). There were no significant differences in the proportion of individuals who had seen a primary care physician, but those in cluster 4 were significantly more likely to have seen a gastroenterologist (*p *= 0.003). The proportion of participants with high CPSS scores and VSI scores was significantly higher in clusters 3 and 4; those characterized by higher psychological burden (*p *< 0.001). In terms of Rome IV functional bowel disorder, >50% of individuals in cluster 2 met criteria for functional diarrhea, and >40% of those in cluster 4 had unspecified functional bowel disorder, but most of the other clusters consisted of a mixture of functional bowel disorder diagnoses. Finally, when we applied our earlier seven cluster solution derived from the 811 participants with Rome IV‐defined IBS to these individuals according to their cluster, 75% met criteria for IBS clusters 2 or 3 (those with low overall gastrointestinal symptom severity with either high or low psychological burden) and a further 15% cluster 1 (those with diarrhea and urgency with low psychological burden; Box 1). More than 50% of those in the second of the five functional bowel disorder clusters would have fallen into cluster 1 of the IBS clusters.

### 3.3 Follow‐up of the different clusters

In total, 330 (59.1%) individuals were successfully followed up at 12 months. Characteristics of those responding to the 12‐month questionnaire, compared with those who did not are provided in Table 2. Responders were more likely to be White Caucasian (*p *< 0.001), and to have seen a primary care physician (*p *= 0.004). They were also less likely to have high levels of gastrointestinal symptom‐specific anxiety or perceived stress at baseline (*p *= 0.008 and *p *= 0.007, respectively). There were no other significant differences.

**TABLE 2:** Characteristics of individuals with Rome IV functional bowel disorders responding to the 12‐month questionnaire compared with non‐responders

	Responded to questionnaire at 12 months (*n* = 330)	Did not respond to questionnaire at 12 months (*n* = 228)	*p* Value*
Mean age (SD)	52.8 (14.3)	49.9 (16.8)	0.031
Female gender (%)	272 (82.4)	183 (80.3)	0.52
Married or co‐habiting (%)	226 (68.5)	143 (62.7)	0.16
University or postgraduate level of education (%)	168 (50.9)	103 (45.6)	0.22
White Caucasian ethnicity (%)	321 (97.3)	204 (89.5)	<0.001
Seen a primary care physician with symptoms at baseline (%)	315 (95.5)	203 (89.0)	0.004
Seen a gastroenterologist with symptoms at baseline (%)	180 (54.5)	112 (49.1)	0.21
HADS anxiety categories at baseline (%)			
Normal	136 (41.2)	89 (39.0)	
Borderline abnormal	75 (22.7)	42 (18.4)	0.25
Abnormal	119 (36.1)	97 (42.5)	
HADS depression categories at baseline (%)			
Normal	230 (69.7)	140 (61.4)	
Borderline abnormal	57 (17.3)	45 (19.7)	0.090
Abnormal	43 (13.0)	43 (18.9)	
PHQ‐12 severity at baseline (%)			
Minimal	36 (10.9)	29 (12.7)	
Low	123 (37.3)	84 (36.8)	
Medium	134 (40.6)	82 (36.0)	
High	37 (11.2)	33 (14.5)	0.52
VSI tertiles at baseline (%)			
Low	156 (47.3)	111 (48.7)	
Medium	119 (36.1)	59 (25.9)	
High	55 (16.7)	58 (25.4)	0.008
CPSS tertiles at baseline (%)			
Low	146 (44.2)	77 (33.8)	
Medium	120 (36.4)	83 (36.4)	
High	64 (19.4)	68 (29.8)	0.007
Rome IV functional bowel disorder at baseline (%)			
Functional constipation	41 (12.4)	35 (15.4)	
Functional diarrhea	121 (36.7)	77 (33.8)	
Functional abdominal bloating or distension	77 (23.3)	52 (22.8)	0.75
Unspecified functional bowel disorder	91 (27.6)	64 (28.1)	
Functional bowel disorder cluster at baseline (%)			
Cluster 1	103 (31.2)	67 (29.4)	
Cluster 2	90 (27.3)	49 (21.5)	
Cluster 3	68 (20.6)	50 (21.9)	
Cluster 4	53 (16.1)	37 (16.2)	
Cluster 5	16 (4.8)	25 (11.0)	0.063

*p value for independent samples *t*‐test for continuous data and Pearson *χ* ^2^ for comparison of categorical data.

At 12‐month follow‐up the proportion of individuals reporting that their symptoms impacted on activities of daily living ≥50% of the time was significantly higher among those with abdominal pain relieved by defecation, loose/watery stools, and urgency (cluster 2), as well as both clusters with above‐average scores for extra‐intestinal and mood‐related symptoms (clusters 3 and 4; *p *< 0.001; Table 3). Those in clusters 3 and 4 were significantly more likely to have consulted a primary care physician or gastroenterologist with their symptoms (*p *< 0.001 for both). Although there was no significant difference in the proportion of individuals commencing a new treatment for their symptoms during follow‐up, the mean number of treatments commenced was significantly higher among those in clusters 3 and 4, versus 1 and 2 (*p *= 0.004). Proportions of participants with abnormal HADS or PHQ‐12 scores were significantly higher among those in clusters 3 and 4 at follow‐up (*p *< 0.001 for all analyses). Finally, those in clusters 3 and 4 were significantly more likely to have experienced a fluctuation of symptoms, such that they had IBS at 12‐month follow‐up (*p *< 0.001), with approximately 50% of individuals in each of these clusters meeting the Rome IV criteria for IBS 12 months later. However, even in clusters 1 and 2, almost one‐third of individuals met Rome IV criteria for IBS at 12‐month follow‐up.

**TABLE 3:** Natural history of the five clusters observed among 330 individuals with a Rome IV functional bowel disorder followed up at 12 months

	Cluster 1 Abdominal pain relieved by defecation and bloating with low psychological burden (*n* = 103)	Cluster 2 Abdominal pain relieved by defecation, loose/watery stools, and urgency with low psychological burden (*n* = 90)	Cluster 3 Abdominal pain, abnormal stool frequency or consistency, urgency, fecal incontinence, and bloating with high psychological burden (*n* = 68)	Cluster 4 Abdominal pain, abnormal stool consistency, urgency, fecal incontinence, and bloating with high psychological burden (*n* = 53)	Cluster 5 Hard/lumpy stools and fecal incontinence with low psychological burden (*n* = 16)	Total (*n* = 330)	*p* Value
Symptoms limiting activities ≥50% of the time at follow‐up (%)	29 (28.2)	41 (45.6)	35 (51.5)	34 (64.2)	1 (6.3)	140 (42.4)	<0.001
Seen a primary care physician regarding IBS during follow‐up (%)	26 (25.2)	20 (22.2)	37 (54.4)	24 (45.3)	4 (25.0)	111 (33.6)	<0.001
Seen a gastroenterologist regarding IBS during follow‐up (%)	12 (11.7)	9 (10.0)	14 (20.6)	21 (39.6)	0 (0)	56 (17.0)	<0.001
Any new treatment commenced during follow‐up (%)	63 (61.2)	46 (51.1)	48 (70.6)	37 (69.8)	10 (62.5)	204 (61.8)	0.090
Mean number of new treatments commenced during follow‐up (SD)	0.97 (0.98)	0.98 (1.15)	1.54 (1.40)	1.49 (1.34)	1.31 (1.40)	1.19 (1.22)	0.004
Abnormal HADS anxiety score at 12‐month follow‐up (score of ≥11) (%)	31 (30.1)	22 (24.4)	34 (50.0)	26 (49.1)	3 (18.8)	116 (35.2)	<0.001
Abnormal HADS depression score at 12‐month follow‐up (score of ≥11) (%)	8 (7.8)	7 (7.8)	13 (19.1)	18 (34.0)	0 (0)	46 (13.9)	<0.001
High levels of somatization at 12‐month follow‐up (score of ≥13) (%)	7 (6.8)	21 (23.3)	41 (60.3)	32 (60.4)	1 (6.3)	102 (30.9)	<0.001
Met Rome IV criteria for IBS at follow‐up (%)	30 (29.1)	26 (28.9)	33 (48.5)	27 (50.9)	1 (6.3)	117 (35.5)	<0.001

*p value for one‐way ANOVA for continuous data and Pearson *χ* ^2^ for comparison of categorical data.

## 4 DISCUSSION

We have derived and validated a prior latent class model for classifying people with Rome IV‐defined IBS into seven unique clusters based on their pattern of gastrointestinal symptoms, extra‐intestinal symptoms, and psychological profiles.^14^ However, although all of the individuals recruited to that study self‐identified as having IBS, only around 60% met Rome IV criteria for IBS, the remainder meeting criteria for another Rome IV functional bowel disorder. We therefore derived a separate latent class model in this group and, by comparing this to our previous IBS model, sought to explore whether the other functional bowel disorders are truly distinct entities or are better conceptualized as a continuum of gastrointestinal illness that includes IBS. We found five unique clusters distinguished by the pattern of gastrointestinal symptoms, extra‐intestinal symptoms, and mood‐related symptoms. Clusters 3 and 4, characterized by high levels of psychological burden, also had significantly higher levels of perceived stress and gastrointestinal symptom‐specific anxiety. Together with cluster 2, defined as abdominal pain not relieved by defecation, diarrhea, and urgency, these clusters also had a significantly higher proportion of people reporting that their symptoms impacted on activities of daily living ≥50% of the time at 12‐month follow‐up. People in clusters 3 and 4 were also significantly more likely to have consulted a doctor about their symptoms and commenced a significantly higher mean number of treatments. This is in keeping with our previous findings in IBS, where clusters with high psychological burden were associated with a poorer prognosis.^19^ They were also significantly more likely to experience a fluctuation of their symptoms such that 50% met criteria for IBS at 12 months. Generally, most of the clusters consisted of a mixture of functional bowel disorder diagnoses, although >50% of individuals in cluster 2 met criteria for functional diarrhea, and >40% of those in cluster 4 had unspecified functional bowel disorder. Application of our previous IBS model showed that three‐quarters of people were assigned to one of two IBS clusters characterized by low overall gastrointestinal symptom severity with either high‐ or low‐psychological burden.

This study recruited a large number of individuals from a community setting who self‐identified as having IBS, of whom a substantial subset did not meet Rome IV criteria for IBS, but instead met Rome IV criteria for another functional bowel disorder. The majority had seen a primary care doctor, some a gastroenterologist, and some had never sought medical advice. It is therefore likely that this cohort, and the latent class model derived from their data, is the representative of people with functional bowel symptoms in general. Nonetheless, participants were identified from a cohort of people who believed that they had IBS, rather than having been diagnosed with another functional bowel disorder directly, and this might limit generalizability of our findings. However, it is debatable whether these other diagnostic labels are frequently used outside of a subspecialized gastroenterology setting. It seems probable that colloquial use of the term “IBS” to describe all functional bowel disorders is common practice for many physicians, particularly given the overlapping symptoms between conditions. Finally, our questionnaire was administered using a web‐based portal meaning that, at both baseline and 12‐month follow‐up, data collection for most variables of interest was complete.

Unfortunately, we were unable to access medical records for participants in this study and were therefore unable to confirm a diagnosis of a functional bowel disorder. Instead, because those participating met Rome IV criteria, we assumed that this was the correct diagnosis. It is important to acknowledge that some organic diseases, such as coeliac disease or inflammatory bowel disease, can mimic IBS and functional bowel symptoms^30^, ^31^, ^32^, ^33^; however, the prevalence of these diseases in the community is much lower. Moreover, most of the participants reported having consulted a doctor regarding their symptoms, and it is therefore plausible that the majority had undergone some investigations, in addition to a clinical assessment, to rule out organic pathology and establish a functional cause for their gastrointestinal symptoms. It is unclear to what extent the cluster profiles derived at baseline and the natural history of the clusters over 12‐month follow‐up may have been influenced by medications taken by the participants to treat their symptoms. The response rate to the 12‐month questionnaire was 59%, which is similar to other longitudinal follow‐up studies of gastrointestinal disorders conducted over a similar time frame.^34^, ^35^, ^36^, ^37^, ^38^ Responders were more likely to be White Caucasian, to have seen a primary care physician, and to have high levels of gastrointestinal symptom‐specific anxiety or perceived stress at baseline. Consequently, the population we studied at follow‐up may not be representative of the original cohort, we recruited; however, the absolute differences between the two groups were relatively modest. Moreover, comparison between responders and the original study participants in terms of psychological comorbidity, baseline cluster membership, and baseline Rome IV functional bowel disorder diagnosis revealed no significant differences.

The functional bowel disorders share the same core gastrointestinal symptoms, namely abdominal pain, change in bowel habit, including both diarrhea and constipation, abdominal bloating, and abdominal distension.^1^ They are differentiated solely according to the relative frequency with which these symptoms are reported, with no compelling evidence of distinct pathophysiological mechanisms separating the conditions. Indeed, they all share a common etiological construct as disorders of gut‐brain interaction.^2^ Consequently, it is debatable whether they are truly discrete disorders, or instead represent a spectrum of gastrointestinal illness. This possibility is emphasized by the observation that relatively minor changes in symptom frequency can change the diagnosis for any individual patient. In the absence of organic disease, a patient who has diarrhea, but who reports pain less than weekly, will meet criteria for functional diarrhea. However, should the frequency of their pain increase to at least weekly, they will instead be diagnosed with IBS with diarrhea (IBS‐D). The same is true of the relationship between symptom reporting and diagnosis in functional constipation compared with IBS‐C. Similarly, bloating and distension are frequently reported by patients with IBS but are not essential to make the diagnosis. Conversely, a patient with functional abdominal bloating and distension might also experience some abdominal pain and/or altered bowel habit, albeit at a threshold insufficient to meet criteria for IBS, functional diarrhea, or functional constipation. Finally, those individuals with unspecified functional bowel disorder can experience any or all of these symptoms whilst not meeting criteria for any other functional bowel disorder. Overall, it could therefore be suggested that people with functional diarrhea, functional constipation, functional abdominal bloating and distension, and unspecified functional bowel disorder are better characterized as suffering from a milder form of IBS.

The data in this study support this hypothesis. Firstly, by applying our previous latent class model for classifying IBS, we found that 75% of people with functional bowel disorders in the present study were in IBS clusters 2 or 3, characterized by low overall gastrointestinal symptoms with high or low psychological burden, respectively. This indicates that most people meeting Rome IV criteria for functional bowel disorders other than IBS are nested within the milder end of the IBS illness spectrum. Second, 50% of those in functional bowel disorder cluster 2 (abdominal pain relieved by defecation, loose/watery stools, and urgency with low psychological burden) were in IBS cluster 1 (diarrhea and urgency with low psychological burden) when we applied our previous IBS latent class model. This suggests that functional diarrhea is actually a less painful IBS‐D phenotype, and indeed, cluster 2 is defined by abdominal pain frequency that is above‐average for this cohort, but which is insufficient to meet criteria for IBS. Moreover, although approximately 60% of those in cluster 2 met criteria for functional diarrhea, all of the clusters consisted of a mixture of Rome IV functional bowel disorder diagnoses indicating that at least some of these may not be real clinical constructs. In cluster 1, although responses relating to alterations in bowel habit are below the cohort average, the fact that 50% of people in this cluster met criteria for either functional diarrhea or functional constipation as shown in Table 1, shows that alterations in bowel habit are still a feature of this cluster. Finally, individuals in clusters 3 and 4 were significantly more likely to experience a fluctuation of symptoms over time. About 50% of these people met Rome IV criteria for IBS at 12 months, as did around one‐third of people in clusters 1 and 2. This reinforces the concept that many of these individuals commence with milder IBS, but that their symptoms worsen over time, an evolution that might, in part, be driven by psychological ill health.

The concept that functional bowel disorders should be treated as a clinical continuum, rather than separate disorders, has important implications for clinical practice and research. It would facilitate a simpler and more pragmatic approach to diagnosis. Studies have shown that primary care physicians rarely apply formal diagnostic criteria for IBS,^39^ yet they are able to diagnose the condition with confidence.^40^ Recently, it has been proposed that the Rome IV criteria for IBS, and other functional bowel disorders, should be modified for use in clinical practice, thereby making them less strict and easier to apply.^41^ The minimum symptom frequency threshold required for diagnosis can be relaxed, provided the cardinal symptoms are present and that they impact on quality of life by being sufficiently bothersome to prompt a consultation with a physician, and the minimum symptom duration of 6 months can be overlooked. This is likely to blur the distinction between IBS and the four other functional bowel disorders even further in clinical practice. As further support for this approach, the present study recruited 1375 individuals who self‐identified as having IBS, of whom over 90% had consulted a primary care doctor with their symptoms, and yet only 60% met Rome IV criteria for IBS, the remainder meeting criteria for another functional bowel disorder. This demonstrates that in everyday clinical practice, and from the patient's perspective, the concept of “IBS” as a clinical entity differs from the definition provided by existing diagnostic criteria. Indeed, given that the first‐line treatment of symptoms across all functional bowel disorders is similar and makes use of dietary modification, antispasmodics, anti‐diarrheals, and laxatives, there may be little practical basis for differentiating them.

Moreover, in the context of clinical trials, any differentiation may be disadvantageous. This is because trials have tended to focus on IBS and functional constipation due to them being more prevalent conditions, resulting in a lack of evidence‐based treatments for the other three functional bowel disorders. Furthermore, although many of the drugs licensed for the treatment of IBS with constipation (IBS‐C)^42^ are also licensed for functional constipation,^43^ this has been achieved by conducting completely separate trials in the two disorders individually, an approach which seems inefficient and unnecessarily expensive. In addition, data suggests that these drugs are effective for treating bloating in IBS‐C,^44^ but none are licensed for treating functional abdominal bloating and distension. Similarly, drugs that are licensed for treating IBS‐D^45^ are not available to treat functional diarrhea as no trials have been conducted in this less prevalent disorder. Finally, the change from the Rome III to Rome IV criteria redefined IBS as an inherently more painful disorder, and patients have more severe symptoms and higher levels of psychological co‐morbidity.^15^ It may therefore be harder to demonstrate the benefit of drugs tested only in those with Rome IV IBS. Recruiting a broader spectrum of patients with different functional bowel disorders of varying severity may limit the extent to which this is an issue.

Consequently, adopting a more pragmatic approach to the conduct of clinical trials in functional bowel disorders, recruiting patients with different, but related, disorders to the same trial, for example, IBS‐D and functional diarrhea, might be preferable.^46^ This would enable treatment to be offered to a wider group of patients, with a wider range of symptom severities, and could facilitate licensing of treatments for different functional bowel disorders simultaneously. It could also make trials more attractive to conduct, make more efficient use of limited research funding,^47^ and simplify recruitment. This may also have the benefit of moving trials away from secondary care settings and into primary care where the vast majority of patients with functional bowel disorders are managed. Results may therefore be a better reflection of real‐world clinical experience, but it would nevertheless still be possible to analyze trial results according to individual Rome IV diagnoses, facilitating comparison of results with previous studies.

In summary, we conducted a LCA in a cohort of people who self‐identified as having IBS, but actually met diagnostic criteria for another Rome IV‐defined functional bowel disorder. We identified five unique clusters differentiated according to the presence of certain gastrointestinal symptoms, including abdominal pain relieved by defecation, bloating, urgency, and altered stool pattern, extra‐intestinal symptoms, and abnormal mood. However, the correlation between these clusters and individual Rome IV functional bowel disorder diagnoses was poor, and when we applied our previous latent class model for Rome IV IBS, three‐quarters of participants would be assigned to one of two IBS subgroups with low overall gastrointestinal symptoms and either high‐ or low‐psychological comorbidity. This suggests that despite meeting criteria for a functional bowel disorder other than IBS, these people might instead be better characterized as suffering from a milder form of IBS. Moreover, at 12‐month follow up, just over one‐third of people reported a change in their symptoms such that they met criteria for Rome IV IBS, and in clusters with high psychological burden, this was even higher at 50%. In keeping with our previous work in IBS, we observed that people in clusters with high psychological burden had a poorer prognosis, being more likely to consult a doctor about their symptoms, to commence a higher mean number of treatments, and to report that their symptoms impacted their daily lives at least 50% of the time. Treating the functional bowel disorders as a spectrum of illness rather than as discrete disorders may help to simply diagnosis and treatment. This is in keeping with recent advice from the Rome Foundation to consider a more pragmatic approach to diagnosis. It could also help to make research trials more inclusive and more attractive to conduct, in turn facilitating wider availability of treatments for patients suffering with often debilitating functional bowel symptoms that remain poorly understood in general, with a frequently substantial impact on overall quality of life.

## AUTHOR CONTRIBUTIONS

CJB, LAH, and ACF conceived and drafted the study. CJB collected all data. CJB and ACF analyzed and interpreted the data. CJB and ACF drafted the manuscript. All authors have approved the final draft of the manuscript.

## CONFLICT OF INTEREST

Christopher J. Black: None to declare. Lesley A. Houghton: None to declare. Alexander C. Ford: None to declare.

## Supporting information

Supplementary Material
