# Supplementary material for: Latent class analysis does not support the existence of Rome IV functional bowel disorders as discrete entities
Source: Neurogastroenterol Motil. 2022 May 9;34(11):e14391. doi: 10.1111/nmo.14391 (PMC9788160; doi:10.1111/nmo.14391)
Supplement: Supplementary file 1 — Supplementary Material [file NMO-34-e14391-s001.docx]

**Supplementary Figure 1. Profiles of the Seven Latent Class Clusters Previously Identified Among 811 People with Rome IV-defined IBS.**


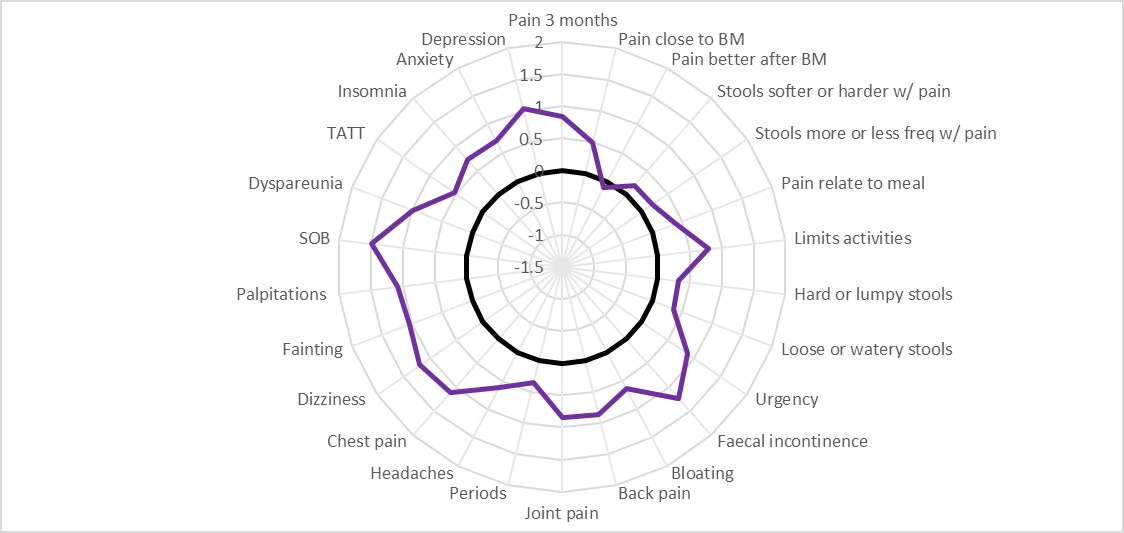

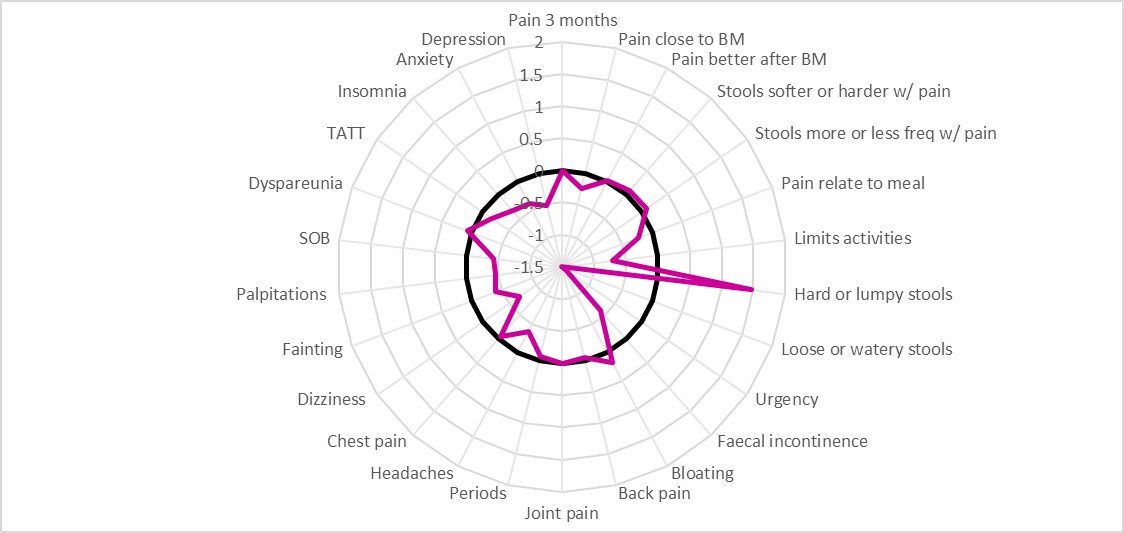

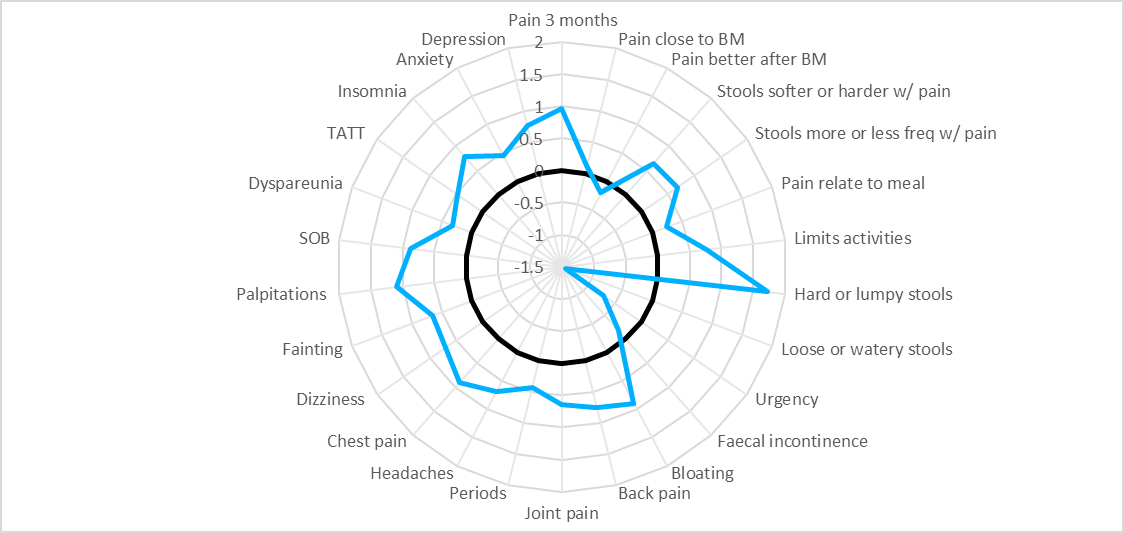


= adjusted cohort mean

**E**

**F**

**G**


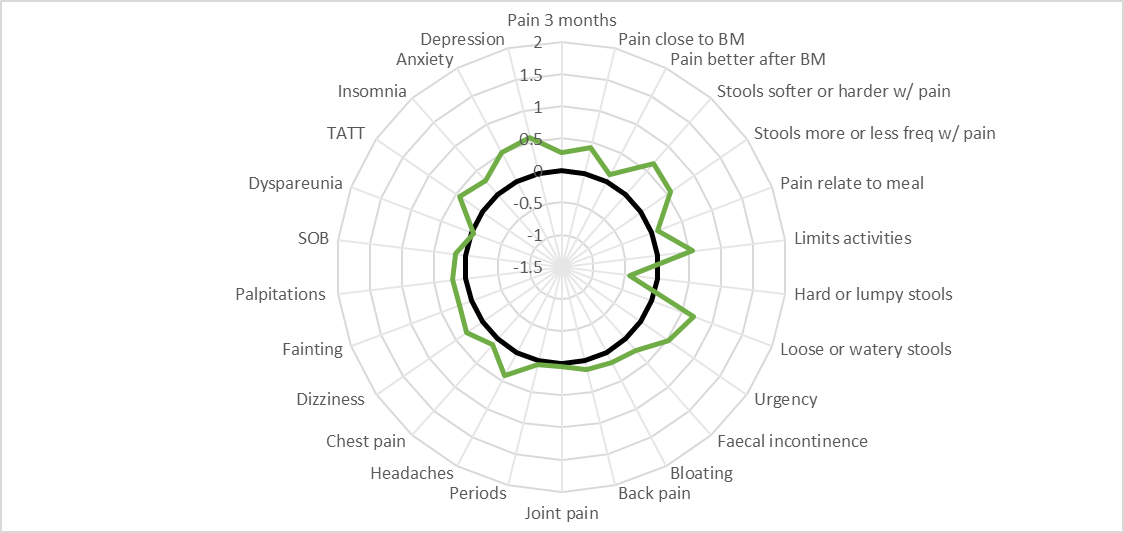

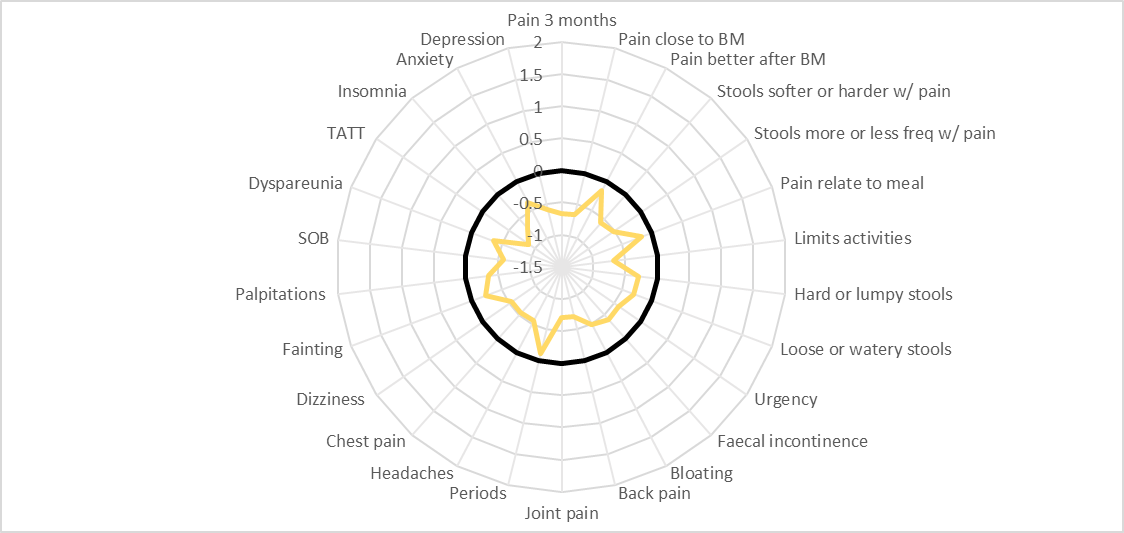

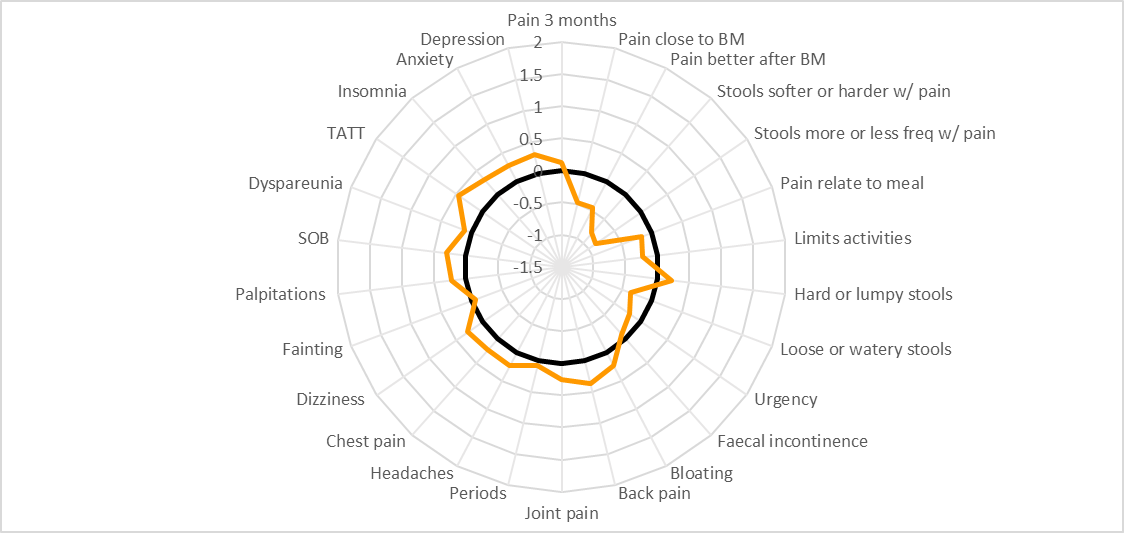

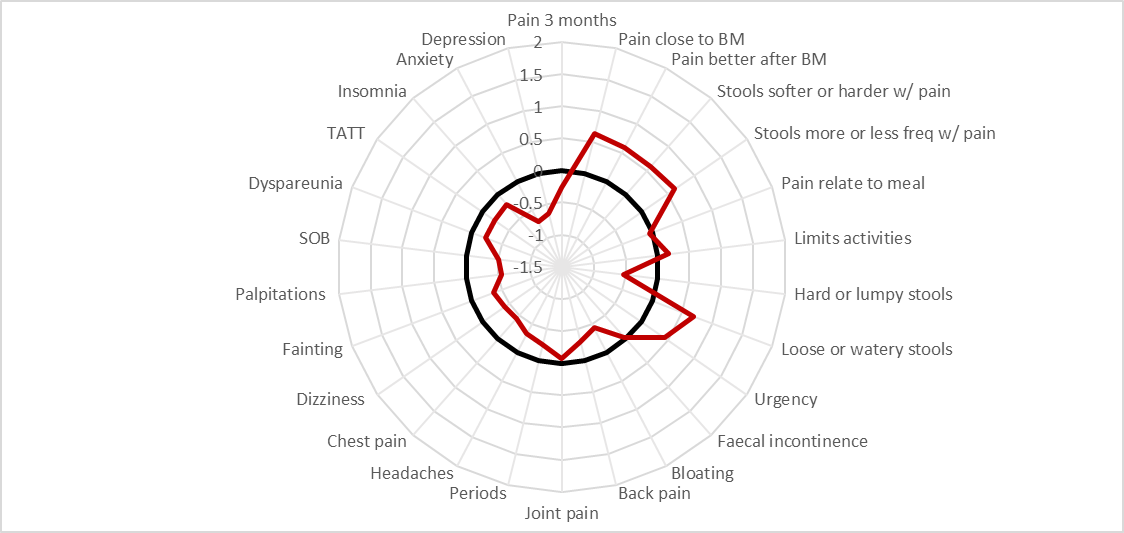


**A**

**B**

**C**

**D**

1. Cluster 1: Diarrhea and urgency with low psychological burden.
2. Cluster 2: Low overall gastrointestinal symptom severity with high psychological burden.
3. Cluster 3: Low overall gastrointestinal symptom severity with low psychological burden.
4. Cluster 4: Diarrhea, abdominal pain, and urgency with high psychological burden.
5. Cluster 5: Constipation, abdominal pain, and bloating with high psychological burden.
6. Cluster 6: High overall gastrointestinal symptom severity with high psychological burden.
7. Cluster 7: Constipation and bloating with low psychological burden.

BM: bowel movement; SOB: shortness of breath; TATT: tired all the time.

**Supplementary Figure 2: Values of BIC(LL) Plotted for Each Specification of the Number of Clusters in the People with a non-IBS Rome IV Functional Bowel Disorder.**

**.**

**Supplementary Table 1. Variables Used in the Latent Class Analysis.**

|  | **Variable** | **Type of variable** | **Scale of Measurement** | **Reason for including in the model** |
| --- | --- | --- | --- | --- |
| **Gastrointestinal Symptoms** | Frequency of abdominal pain (or discomfort*) anywhere in the abdomen in past 3 months | Ordinal | 9-point scale from “Never” (0) to “Multiple times per day or all the time” (8) | All of these variables for quantifying gastrointestinal symptoms were taken from Rome Foundation questionnaires. These are the recognized “gold standard” for diagnosing IBS, and are widely used. |
|  | Frequency of abdominal pain being closely related to a bowel movement | Ordinal | 11-point scale from “0%” (never) to “100%” (always) |  |
|  | Frequency with which abdominal pain improved or resolved following a bowel movement | Ordinal | 11-point scale from “0%” (never) to “100%” (always) |  |
|  | Frequency with which stools became softer or harder than usual in association with abdominal pain | Ordinal | 11-point scale from “0%” (never) to “100%” (always) |  |
|  | Frequency with which stools became more or less frequent than usual in association with abdominal pain | Ordinal | 11-point scale from “0%” (never) to “100%” (always) |  |
|  | Frequency with which abdominal pain started or got worse after a meal | Ordinal | 11-point scale from “0%” (never) to “100%” (always) |  |
|  | Frequency with which abdominal pain restricted usual activities | Ordinal | 11-point scale from “0%” (never) to “100%” (always) |  |
|  | Frequency of hard or lumpy stools in last 3 months | Ordinal | 5-point scale from “0%” (never or rarely) to “100%” (always) |  |
|  | Frequency of loose, mushy, or watery stools in the last 3 months | Ordinal | 5-point scale from “0%” (never o rarely) to “100%” (always) |  |
|  | Frequency of fecal urgency over last 3 months | Ordinal | 9-point scale from “Never” (0) to “Multiple times per day or all the time” (8) |  |
|  | Frequency of fecal incontinence over last 3 months | Ordinal | 9-point scale from “Never” (0) to “Multiple times per day or all the time” (8) |  |
|  | Frequency of abdominal bloating or distension over last 3 months | Ordinal | 9-point scale from “Never” (0) to “Multiple times per day or all the time” (8) |  |
| **Extraintestinal Symptoms** | All individual items of the PHQ-12 and the frequency experienced in the last 4 weeks:  Back pain  Arm, leg, joint pain  Period pain/period problems  Headaches  Chest pain  Dizziness  Fainting spells  Heart pounding/racing  Shortness of breath  Pain/problems during sex  Feeling tired or low in energy  Trouble sleeping | Ordinal | 3-point scale: “Never” (0), “A little” (1), or “A lot” (2) | Reporting symptoms referable to multiple body systems, also referred to as somatization, is recognized as being associated with IBS and other functional GI disorders. The PHQ-12 questionnaire is a widely used and validated method for measuring this. |
| **Mood** | Presence of anxiety, as measured by the total score of the HADS-Anxiety questionnaire | Ordinal | 3-point scale: normal (0), borderline (1), or abnormal (2) | Abnormal mood is well-recognized as being an important factor in IBS. The HADS questionnaire for quantifying the presence of anxiety and/or depression are widely used and validated for this purpose. |
|  | Presence of depression, as measured by the total score of HADS-Depression questionnaire | Ordinal | 3-point scale: normal (0), borderline (1), or abnormal (2) |  |
